# Supplementary material for: Intracellular Toxic Advanced Glycation End-Products in 1.4E7 Cell Line Induce Death with Reduction of Microtubule-Associated Protein 1 Light Chain 3 and p62
Source: Nutrients. 2022 Jan 13;14(2):332. doi: 10.3390/nu14020332 (PMC8777741; doi:10.3390/nu14020332)
Supplement: Supplementary file 1 [file nutrients-14-00332-s001.zip › nutrients-1497584-supplementary.pdf]

nutrients

MDPI

Supplementary Materials

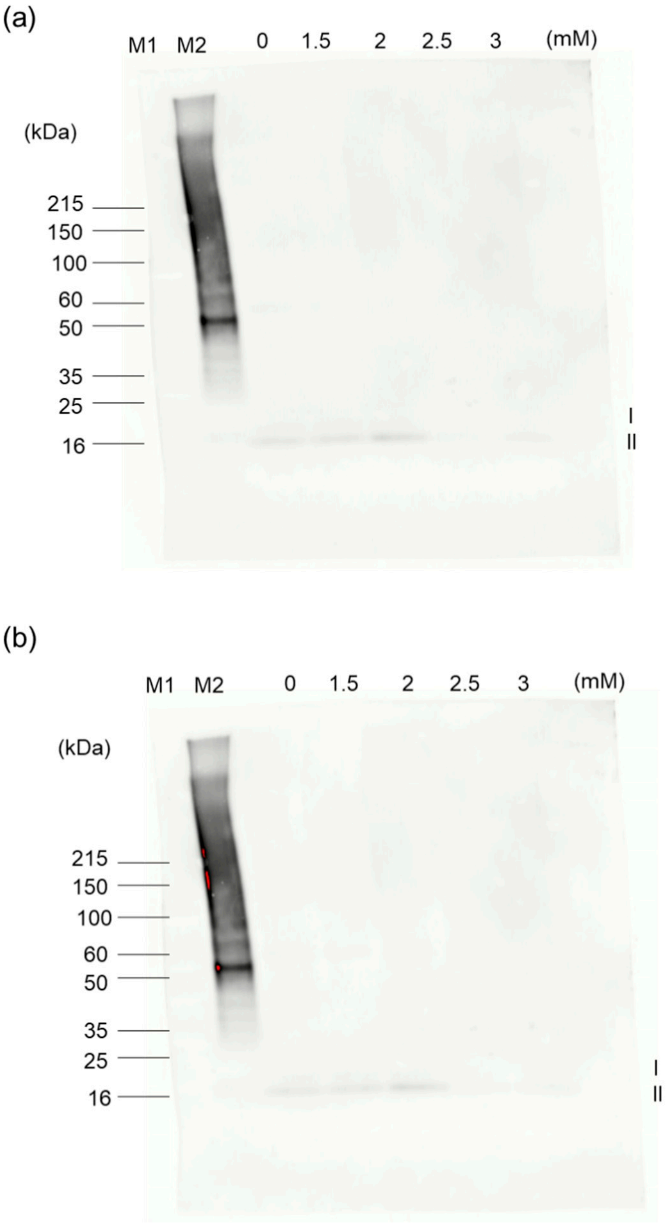

Figure S1. Full-length blots of the LC3 image presented in Figure 2a. Image was obtained following a short exposure time under chemilumi mode. GA: glyceraldehyde. (**a**) The band of LC3 was detected on a polyvinylidene difluoride (PVDF) membrane with full sensitivity and 20 s expose. (**b**) The band of LC3 was detected on a PVDF membrane with full sensitivity and 60 s expose. (**a**,**b**) M1: molecular weight size marker can be to observed under visible light in this lane. M2: HRP-linked molecular weight size marker was loaded in this lane. Molecular weights are indicated by the marker in M2. I and II show the LC3-I and -II position.

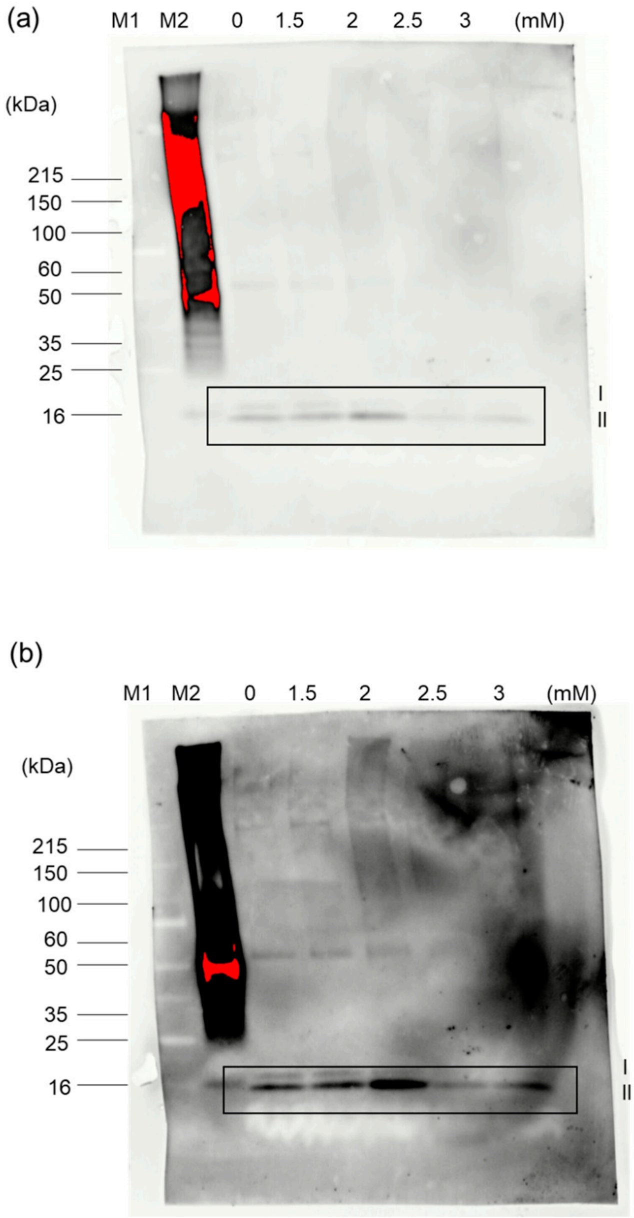

Figure S2. Full-length blots of the LC3 image presented in Figure 2a, which was obtained following a long exposure duration with chemilumi mode. GA: glyceraldehyde. (**a**) The band of LC3 was detected on the PVDF membrane with full sensitivity and 240 s expose. (**b**) The band of LC3 was detected on the PVDF membrane with full sensitivity and 120 s expose, and the value of the gray scale was 5000 in Fusion FX software. (**a**,**b**) M1: molecular weight marker can be observed under visible light in this lane. M2: HRP-linked molecular weight marker was loaded in this lane. The molecular weights are indicated by the marker in M2. I and II show the LC3-I and -II position. The open box replaces the LC3-I and -II bands indicated in Figure 2a.

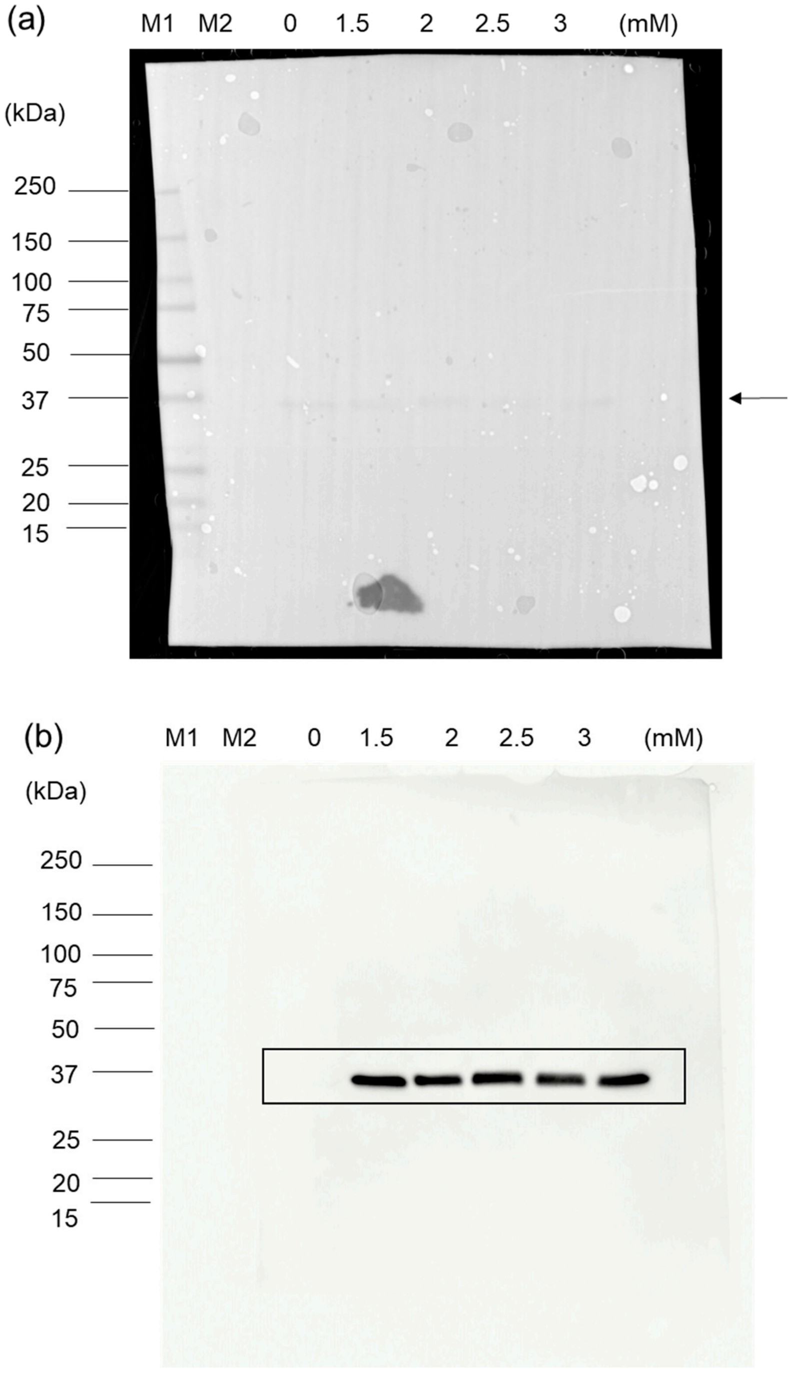

Figure S3. Full-length blots of the GAPDH image presented in Figure 2a. GA: glyceraldehyde. (**a**) The PVDF membrane was exposed for 480 ms in White Epi mode. The arrow indicates the position of the compound obtained using an anti-GAPDH antibody, a secondary antibody, and reagents of the ImmunoStar LD kit. (**b**) The band of GAPDH was detected on the PVDF membrane under chemilumi mode. The open box replaces the GAPDH bands indicated in Figure 2a. (**a**,**b**) M1: molecular weight marker that can be observed under visible light is shown in this lane. M2: HRP-linked molecular weight marker was loaded in this lane. The molecular weights are indicated by the marker in M1.

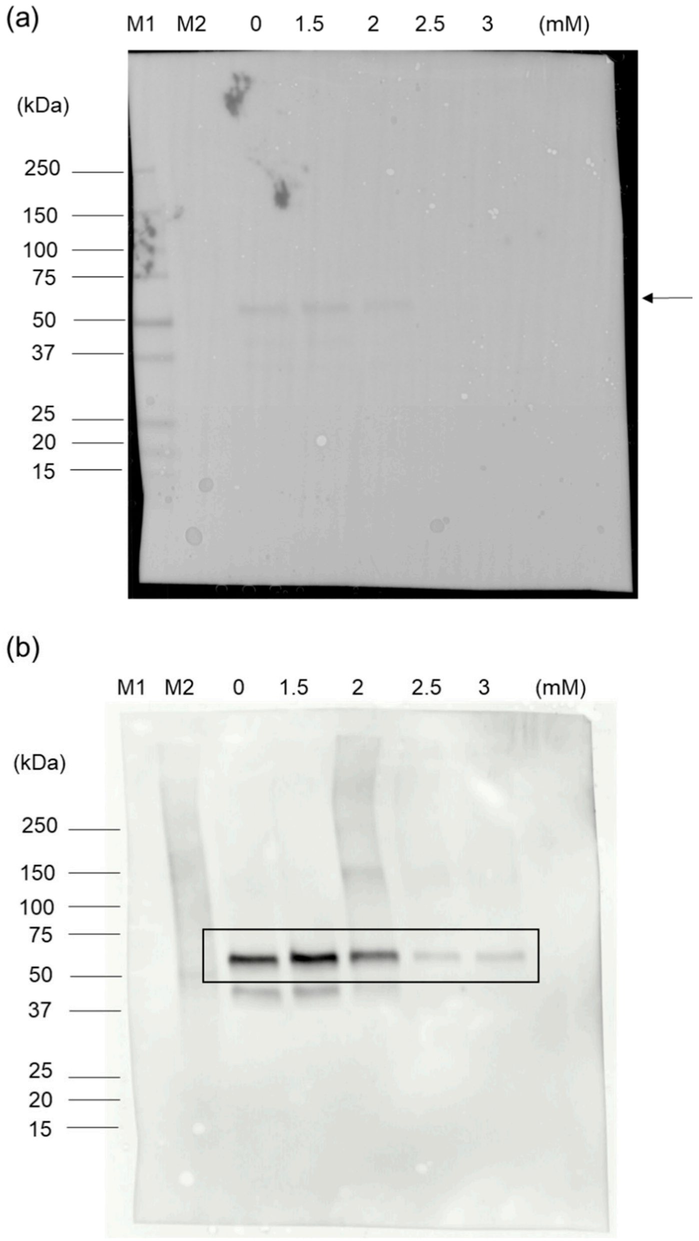

Figure S4. Full-length blots of the p62 images presented in Figure 2a. GA: glyceraldehyde. (**a**) The PVDF membrane was exposed for 480 ms in White Epi mode. The arrow indicates the position of the compound obtained using an anti-p62 antibody, a secondary antibody, and reagents of the ImmunoStar LD kit. (**b**) The band of GAPDH band was detected on the PVDF membrane in chemilumi mode. The open box replaces the p62 bands indicated in Figure 2a. (**a**,**b**) M1: molecular weight marker can be observed under visible light in this lane. M2: HRP-linked molecular weight marker was loaded in this lane. The molecular weights are indicated by the marker in M1.

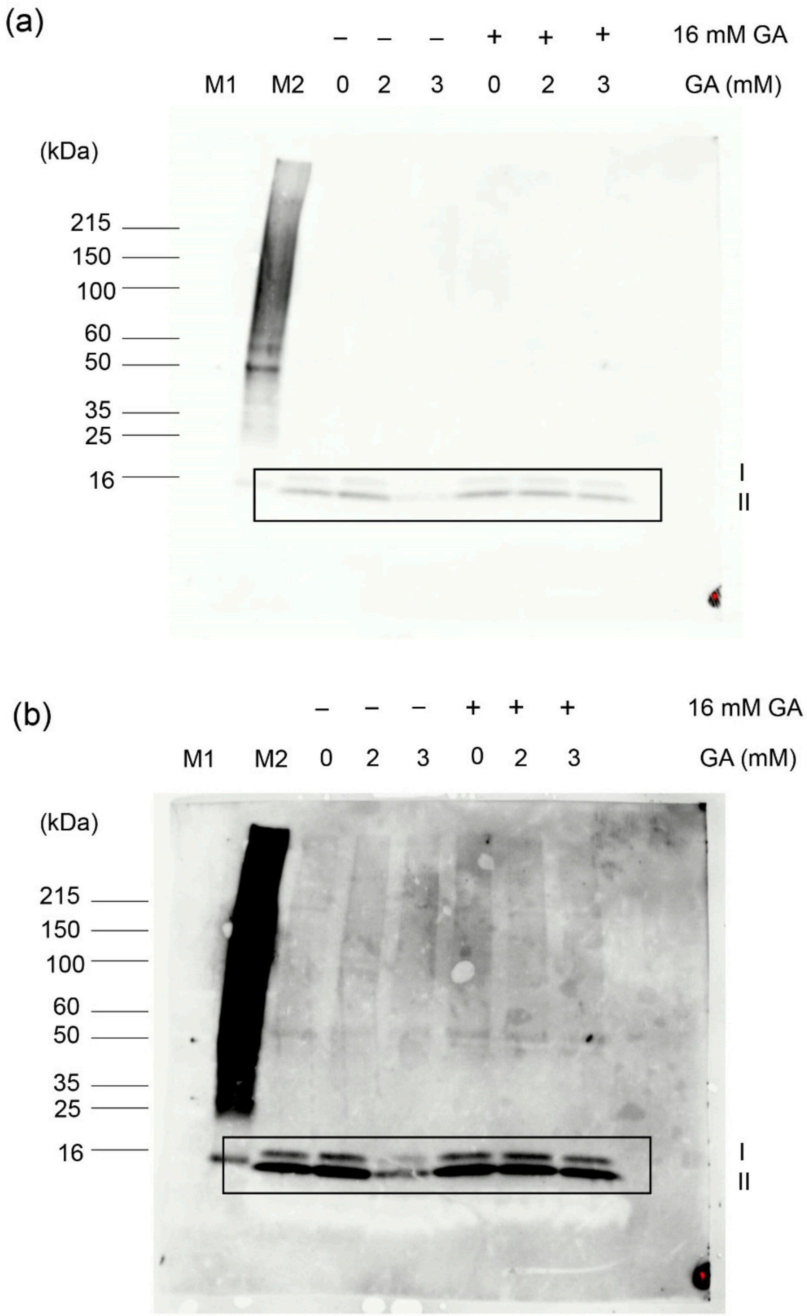

Figure S5. Full-length blots of the LC3 image in Figure 3a, which was obtained following exposure for in chemilumi mode. GA: glyceraldehyde. Ag: aminoguanidine (**a**) The LC3 band was detected on the PVDF membrane with full sensitivity and 120 s expose. (**b**) The LC3 band was detected on the PVDF membrane with full sensitivity and 120 s expose, and the value of the gray scale was 10,000 in the Fusion FX software. (**a**,**b**) M1: molecular weight can be observed under visible light in this lane. M2: HRP-linked molecular weight marker was loaded in this lane. The molecular weights are based on the marker in M1. I and II show the LC3-I and -II position. The open box replaces the LC3-I and -II bands indicated in Figure 2a. The molecular weights are based on the marker size in M2.

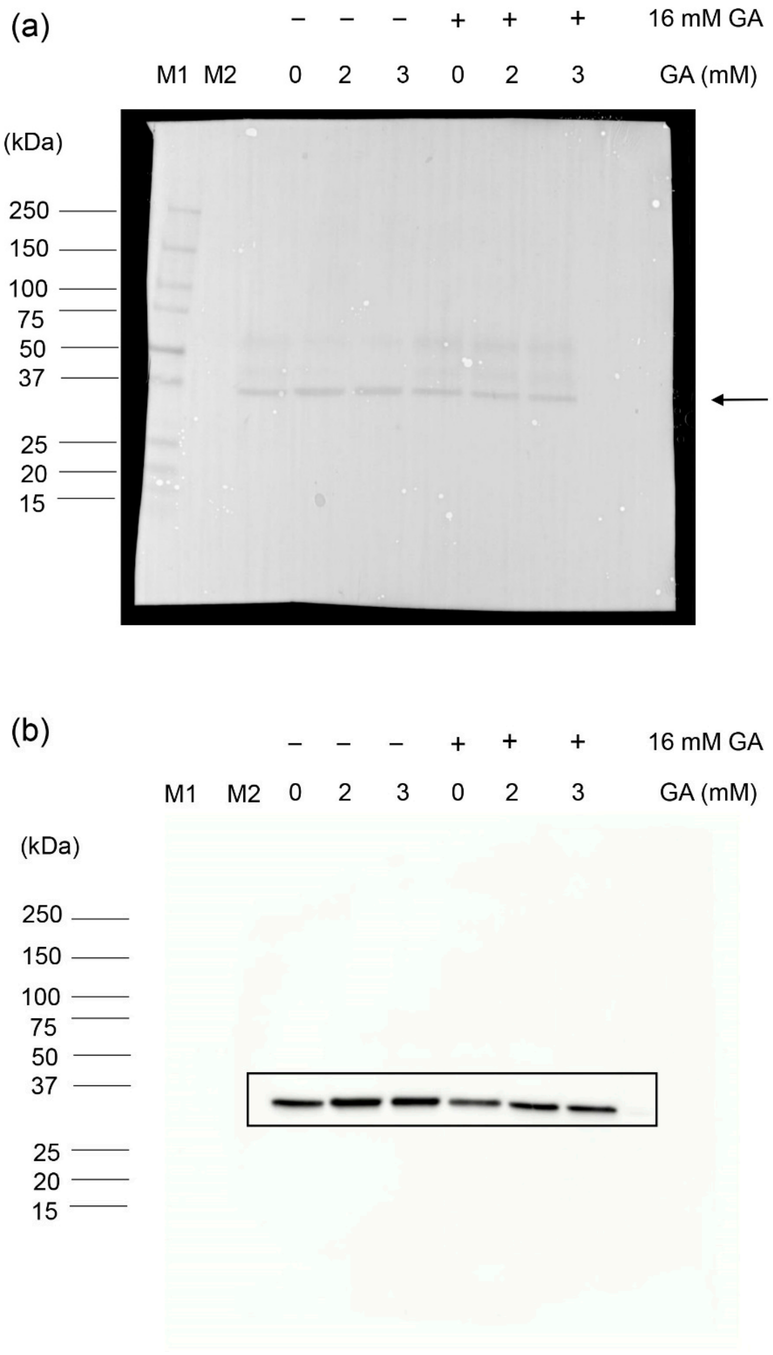

Figure S6. Full-length blots of GAPDH image presented in Figure 3a. GA: glyceraldehyde. AG: aminoguanidine (**a**) The PVDF membrane was exposed for 480 ms in White Epi mode. The arrow indicates the position of the band obtained with an anti-GAPDH antibody, a secondary antibody, and reagents of ImmunosterLD kit. (**b**) The band of GAPDH was detected on the PVDF membrane with chemilumi mode. The open box replaces the GAPDH bands indicated on Figure 3a. (**a**,**b**) M1: molecular weight marker can be observed under visible light in this lane. M2: HRP-linked molecular weight marker was loaded in this lane. The molecular weights are based on the marker in M1.

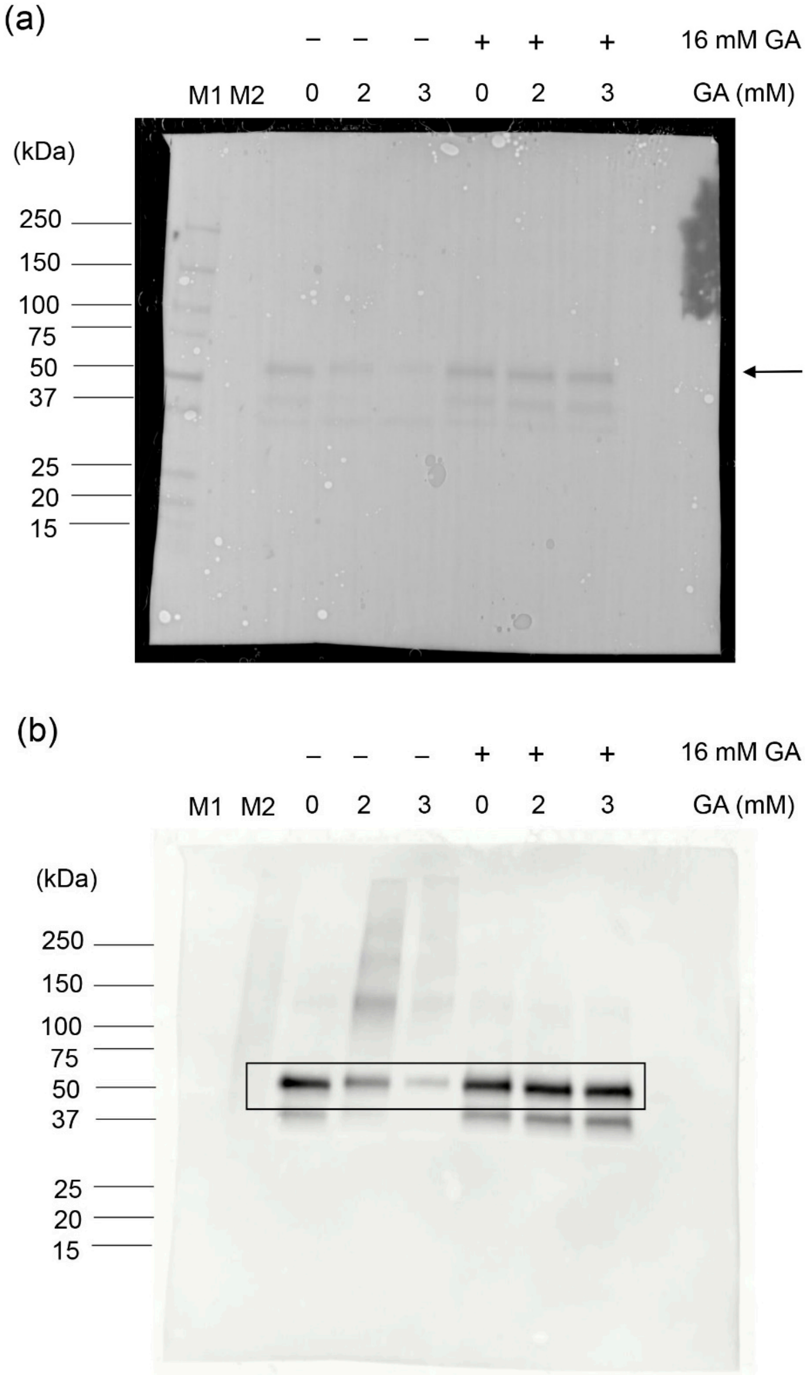

Figure S7. Full-length blots of the p62 image presented in Figure 3a. GA: glyceraldehyde. AG: aminoguanidine (**a**) The PVDF membrane was exposed for 480 ms in White Epi mode. The arrow indicates the position of the band generated by an anti-p62 antibody, a secondary antibody, and reagents of the ImmunoStar LD kit. (**b**) A band p62 was detected on the PVDF membrane under chemilumi mode. The open box replaces the p62 bands indicated in Figure 3a. (**a**,**b**) M1: molecular weight marker can be observed under visible light in this lane. M2: HRP-linked molecular weight marker was loaded in this lane. The molecular weights are located based on the marker in M1.
